# The Fear Reduction Exercised Early (FREE) approach to low back pain: study protocol for a randomised controlled trial

**DOI:** 10.1186/s13063-017-2225-8

**Published:** 2017-10-17

**Authors:** Ben Darlow, James Stanley, Sarah Dean, J. Haxby Abbott, Sue Garrett, Fiona Mathieson, Anthony Dowell

**Affiliations:** 1 Department of Primary Health Care and General Practice, University of Otago, Wellington, New Zealand; 2 Biostatistical Group, Dean’s Department, University of Otago, Wellington, New Zealand; 3 Medical School, University of Exeter, Exeter, UK; 4 Department of Surgical Sciences, University of Otago, Dunedin, New Zealand; 5 Department of Psychological Medicine, University of Otago, Wellington, New Zealand

**Keywords:** Low back pain, RCT, Primary care, General practice, Intervention study, Treatment outcome, Impairment, Brief intervention, Cost-effectiveness, Health-related quality of life

## Abstract

**Background:**

Low back pain (LBP) is a major health issue associated with considerable health loss and societal costs. General practitioners (GPs) play an important role in the management of LBP; however, GP care has not been shown to be the most cost-effective approach unless exercise and behavioural counselling are added to usual care. The Fear Reduction Exercised Early (FREE) approach to LBP has been developed to assist GPs to manage LBP by empowering exploration and management of psychosocial barriers to recovery and provision of evidence-based care and information. The aim of the Low Back Pain in General Practice (LBPinGP) trial is to explore whether patients with LBP who receive care from GPs trained in the FREE approach have better outcomes than those who receive usual care.

**Methods/design:**

This is a cluster randomised controlled superiority trial comparing the FREE approach with usual care for LBP management with investigator-blinded assessment of outcomes. GPs will be recruited and then cluster randomised (in practice groups) to the intervention or control arm. Intervention arm GPs will receive training in the FREE approach, and control arm GPs will continue to practice as usual. Patients presenting to their GP with a primary complaint of LBP will be allocated on the basis of allocation of the GP they consult. We aim to recruit 60 GPs and 275 patients (assuming patients are recruited from 75% of GPs and an average of 5 patients per GP complete the study, accounting for 20% patient participant dropout). Patient participants and the trial statistician will be blind to group allocation throughout the study. Analyses will be undertaken on an intention-to-treat basis. The primary outcome will be back-related functional impairment 6 months post-initial LBP consultation (interim data at 2 weeks, 6 weeks and 3 months), measured with the Roland-Morris Disability Questionnaire. Secondary patient outcomes include pain, satisfaction, quality of life, days off from work and costs of care. Secondary GP outcomes include beliefs about pain and impairment, GP confidence, and actual and reported clinical behaviour. Health economic and process evaluations will be conducted.

**Discussion:**

In the LBPinGP trial, we will investigate providing an intervention during the first interaction a person with back pain has with their GP. Because the FREE approach is used within a normal GP consultation, if effective, it may be a cost-effective means of improving LBP care.

**Trial registration:**

Australian New Zealand Clinical Trials Registry, ACTRN12616000888460. Registered on 6 July 2016.

**Electronic supplementary material:**

The online version of this article (doi:10.1186/s13063-017-2225-8) contains supplementary material, which is available to authorized users.

## Background

Low back pain (LBP) is a highly prevalent and expensive health condition [1–4]. At any given point in time, 18% of the world’s population may be experiencing LBP, and 38% of people will experience LBP over the course of 1 year [5]. Back pain is one of the leading causes of health loss globally and in New Zealand [6, 7].

Back pain has been estimated to cost 2% of gross domestic product in developed countries [3, 4]. Health care use represents about 15% of the total societal cost of LBP, with the majority of costs due to absence from work and decreased productivity [2]. Consequently, interventions that promote early return to work and that minimise lost productivity are likely to have the greatest impact on the societal burden of LBP [2].

Back pain is a very common reason to visit general practitioners (GPs), both internationally and in New Zealand [1, 8, 9]. People are more likely to seek care when they have high levels of disability and/or pain [10, 11]. Although GP care is cheaper than other treatments for LBP, authors of a systematic review found that it is generally less cost-effective when costs associated with loss of earnings and changes in productivity are taken into account [12]. However, when exercise and behavioural counselling were added to usual GP care, it became the most cost-effective approach [12].

Factors that predict persistent LBP-related activity or work limitation include high initial levels of pain and impairment, psychiatric co-morbidities, low health status and a range of psychosocial factors [13, 14]. Low pain self-efficacy beliefs, poor expectation of recovery, elevated fear avoidance beliefs, catastrophisation, psychological distress (anxiety, depression and stress) and reliance on passive coping strategies have all been found to be independently associated with poor disability outcomes [15–23]. Those who have more maladaptive beliefs, particularly higher levels of fear or more catastrophic beliefs, are also more likely to seek care [10, 24, 25].

The Fear Reduction Exercised Early (FREE) approach was developed in New Zealand to assist GPs manage LBP [26]. This complex intervention includes GP training, electronic consultation support and patient information resources. Its aim is to empower GPs to explore and address psychosocial barriers to recovery and provide evidence-based care and information to their patients with LBP. FREE may be delivered by a GP at the first consultation a person has for an episode of acute LBP or during subsequent LBP consultations. Researchers found in pilot testing that the FREE approach was acceptable to GPs and considered to be useful [26]. The aim of this study is to explore whether patients with LBP who receive care from GPs who are trained in the FREE approach have better outcomes than those who receive usual care.

## Methods/design

### Aim

The primary objective of the Low Back Pain in General Practice (LBPinGP) Study is to measure the effectiveness of the FREE approach versus usual care for LBP in terms of reduction in back-related functional impairment. Secondary objectives are to (1) measure the effectiveness of the FREE approach versus usual care for LBP in terms of reduction in pain and increase in health-related quality of life and patient satisfaction; (2) measure the cost-effectiveness of the FREE approach and usual care over a period of 6 months from societal, health system and Accident Compensation Corporation (ACC; a New Zealand government controlled universal accident insurance scheme) perspectives; and (3) consider which elements of the intervention mediate any observed changes in health outcomes. In sensitivity analysis, we will explore these same outcomes for new episodes of acute LBP (defined as less than 6 weeks’ duration with no LBP-related care received during the preceding 3 months) to explore the study’s initial hypothesis that FREE will be most effective when received at the first consultation for an episode of acute LBP.

### Design

This study is a cluster randomised controlled superiority trial comparing the FREE approach to usual care for LBP management with investigator-blinded assessment of outcomes. The Standard Protocol Items: Recommendations for Interventional Trials (SPIRIT) checklist is available as an Additional file 1.

### Setting of the study

This study will be based in one geographical region of New Zealand that has a population base of 144,550 people. Compared with the New Zealand general population, the study population has a slightly higher proportion of Māori and Pacific people as well as similar proportions of people in each level of socioeconomic deprivation [27]. Patients will be recruited from general practice clinics in this region, which has a total of 21 practices.

### Participants

#### General practices

Up to ten general practices will be recruited from the study region. Practices in the region which have more than three full-time equivalent (FTE) GPs will be invited to participate in the trial in collaboration with their primary healthcare organisations (PHOs). Practices will not be invited if the PHO considers that trial participation will place an unacceptable burden due to other factors. Recruitment will not be feasible in practices smaller than three FTE GPs, given the low relative incidence of LBP presentations. Practices will enter the trial in pairs because sequential participation of practices will permit close contact with practices to optimise patient recruitment. The sequence of participation will be planned in collaboration with practices to enable simultaneous recruitment from pairs of intervention and control practices that are of similar size.

#### General practitioners

**Fig. 1:**
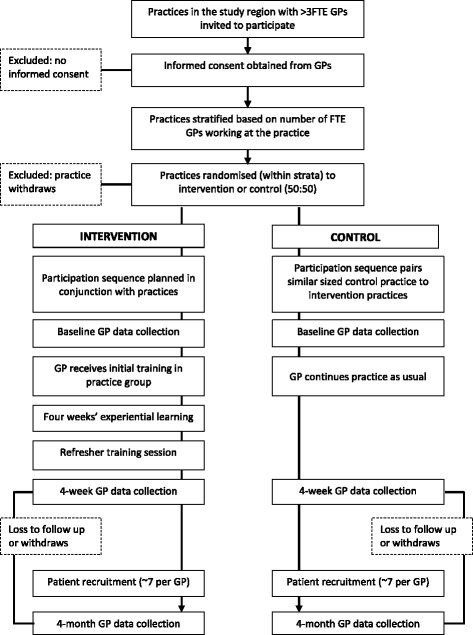
General practitioner (GP) trial process. *FTE* Full-time equivalent

GPs will be invited to participate if they are registered medical practitioners working at a general practice in the study region that has consented to participate in the trial. GPs will be excluded if they have participated in pilot testing of the FREE approach. GP flow through the trial is presented in Fig. 1. GPs invited to participate will receive a participant information sheet. Written informed consent will be obtained by members of the research team.

#### Patients

##### Screening

**Fig. 2:**
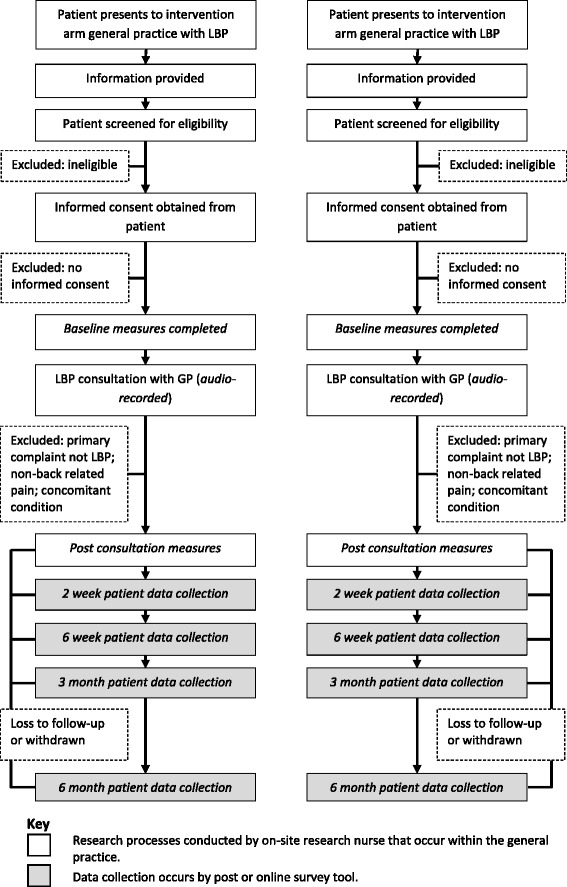
Patient participant trial processes. *GP* General practitioner, *LBP* Low back pain

All patients presenting to participating GPs with a primary complaint of LBP will be screened for participation by a research nurse following a brief explanation of the study. Patients meeting all inclusion criteria and no exclusion criteria will be eligible for enrolment. Patients will be enrolled and complete baseline surveys before they see their GP. Patient flow through the trial is presented in Fig. 2.

##### Inclusion/exclusion criteria

This study will include patients aged 18 years and over who present to a participating GP with LBP of any duration as their primary complaint. Patients will not be eligible if they have had back surgery in the last 6 months, have been unable to do their normal work (or normal daily activities for those who are retired, unemployed or work at home) for more than 3 of the last 6 months, have LBP due to a non-back-related condition (e.g., cauda equina compression, inflammatory arthritis, malignancy, infection, aortic stenosis or aneurysm), have a concomitant health condition which means they are not suitable for trial participation (e.g., pregnancy or major psychological disturbance), or are unable to read and write in English.

##### Informed consent

This trial complies with the Declaration of Helsinki and has received approval from the New Zealand Central Region Health and Disability Ethics Committee (16/CEN/43). Because the FREE approach is consistent with current best practice recommendations [28], patients do not need to consent to receive treatment with the approach itself. Patients will need to consent to being a research participant, but this process will not refer to the type of treatment they may receive. Eligible patients will receive a participant information sheet. Written informed consent will be obtained by a research nurse.

##### Concomitant care

Patient care concomitant to the FREE intervention will not be controlled or limited. It will be monitored through the Otago Costs and Consequences Questionnaire for Low Back Pain (OCC-Q-LBP).

### Participant retention

During the trial, contact will be maintained with practices by having a research nurse on site or on call during patient enrolment, as well as regular emails and monthly practice visits from the research team. Patients will be sent a voucher to acknowledge their participation in each questionnaire round. Vouchers will be delivered with the subsequent questionnaire (so that participants will receive a voucher at the same time as a questionnaire) to maximise responses [29, 30]. Methods of follow-up may include text message, email, telephone and social media [31], with participants consenting to follow up by one or more of these methods at enrolment. A website (www.lowbackpain.co.nz) will also be used to communicate with participants and for participants to advise researchers of changes to contact details. A further, optional consent will be obtained to follow participants for up to 5 years through subsequent surveys using the same instruments employed in the present study.

### Allocation arms

#### Intervention group

GPs in practices allocated to the intervention group will be trained in the FREE approach. The FREE approach to LBP was developed on the basis of a systematic review of the literature [32], in-depth interviews with GPs and people who had acute and chronic LBP [33–35], and a national survey [36]. These findings were synthesised with current research evidence on back pain, behaviour change and guideline implementation to create a novel approach. A full description of the development and basis of FREE is available elsewhere [26].

The FREE approach is a complex intervention which combines GP and patient belief change with behaviour change approaches. The aim of FREE is to optimise the behaviour of both GPs and patients and shift focus to factors and behaviours which have been shown to influence outcome while reducing the provision of unhelpful, threat-related information to patients.

Key GP behaviour change goals are (1) increased confidence to manage back pain and reduced GP anxiety related to screening for pathology; (2) increased understanding of the impact of psychosocial factors on patient outcomes, with tools for exploring and managing these factors; and (3) increased confidence that movement and activity are safe, with tools for demonstrating this confidence to patients. Key patient behaviour change goals are (1) reduced threat associated with LBP, (2) decreased fear of movement and decreased perceived need to protect the back, (3) improved expectation of outcome from a back pain episode, and (4) increased activity and work participation.

Additional file 2 describes the behaviour change techniques (BCTs) employed in FREE in relation to GPs. The BCTs employed for patients are presented in Additional file 3. These techniques are described using the BCT taxonomy developed by Michie et al. [37].

GPs will be trained in the FREE approach through a 4-h facilitated workshop supported with a training manual. GPs then use the approach during a 4-week experiential learning period. It is anticipated that GPs will see at least three patients with LBP during the learning period. GPs will then attend a 1-h refresher session to discuss experiences and resolve any implementation difficulties. The same investigator (BD) will lead all GP training workshops and refresher sessions. Patient enrolment in the study will commence after this refresher session. The approach also includes an electronic medical record (EMR) tool to facilitate use of the approach, an information booklet for patients and GPs, and an information website accessible by both patients and GPs.

#### Control group

GPs within a practice allocated to the control group will receive no training during the intervention and follow-up periods, during which time they will continue to provide current usual care. This has been chosen as the comparator because it is the best care available locally outside the study. All GPs have access to current LBP treatment guidelines.

At the end of the trial, control group GPs will be offered an opportunity to attend the FREE workshop. This will provide comparable benefits for being part of a research trial.

### Randomisation, allocation concealment and blinding

#### Randomisation

General practices will be randomly assigned to either the intervention or control group (i.e., all GPs in a practice will be randomised to the same arm) with a 1:1 allocation, using a computer-generated randomisation schedule stratified by the number of FTE GPs within the practice (≤8 versus > 8 FTE). The aim of the stratification procedure is to ensure that approximately equal numbers of GPs are allocated to the intervention and control groups. An independent statistician will conduct the randomisation process. Analysis of outcomes will include this practice size stratification variable as a fixed effect to account for the impact of this design element on study results.

GP participants will be randomised by practice for the following two reasons: (1) Individual patient randomisation is not feasible, because GPs may find it difficult to provide ‘usual care’ after learning about the new approach, which would result in contamination of results from control patients; and (2) randomisation at the level of the individual GP (rather than practice) may result in contamination effects if intervention GPs discuss the FREE approach with control arm GPs within their practice.

#### Allocation concealment

All general practices participating in the trial (and GP participants currently working in these practices) will be recruited before group allocation occurs. GPs may leave and others may join these practices (and become eligible for the trial) during the period between randomisation and data collection. New GPs will be kept blinded to their practice’s group allocation until they have decided whether to participate in the trial (using the same information as their peers). An independent statistician at a central administration site will perform the randomisation. The independent statistician will communicate practice allocation to the primary investigator so that training may be planned in intervention practices and data collection dates can be agreed upon with all practices.

Patient recruitment will occur post-randomisation. All eligible patients will be invited to participate. Patients will be unaware of the trial’s existence and goals prior to presenting at the practice. Patients will also be unaware that two different treatment approaches are being compared within the trial, meaning that patient participants will be masked to cluster allocation. Research nurses at intervention and control group practices will use identical scripts and information sheets when approaching potential participants, screening participants for inclusion and explaining the study to minimise potential recruitment bias.

#### Blinding

The results dataset will be stored with the study arm identity blinded by way of a unique code for each study participant. The key to this code will be held (independent of the dataset for analysis) by the independent statistician, the chair of the data monitoring committee (DMC) and the principal investigator (PI). The PI needs to know practice group allocation to conduct the intervention group training workshops. The PI will not be involved in data collection, entry, modification or analysis.

Individual patients will remain blind to the presence of two study arms throughout the course of the trial. Consultation appearance will be similar from a patient perspective, except that intervention arm participants may be provided a FREE booklet and referred to the FREE website. Control arm participants may be given other informational materials (e.g., ACC pamphlet) or be referred to other information sites (e.g., patient.org). Even if a patient perceives he/she is receiving a different approach from previous GP LBP consultations, they will remain blind to the study hypothesis.

All patient participant data submitted on paper surveys will be entered or checked by a research assistant blind to group allocation. Data entered by participants through electronic surveys will be checked for completeness and anomalous responses.

Data cleaning and analysis will be conducted by the trial statistician who is blind to group allocation. Once analysis is complete, the results will be unblinded. To eliminate the risk that the trial statistician may have been inadvertently exposed to practice codes during the conduct of the trial, these will be replaced with new codes in the dataset prior to the trial statistician’s receiving the data. Only the independent statistician and the chair of the DMC will have access to the key for these new codes.

GPs and practices will necessarily be unblinded following group allocation because the need for training means GPs will know whether they are in the intervention or control arm prior to patient recruitment. GPs will be aware of their study allocation when completing baseline measures. This is necessary in order to minimise the time between completing baseline measures and receiving FREE training or starting patient recruitment. The content of the intervention will be unknown to all GPs at the time they complete their baseline questionnaire.

### Adverse events

The risks to participants in this trial are small, and no serious adverse events or side effects attributable to the treatment provided are expected. Patients and GPs will be encouraged to report all potential adverse events and incidents of serious pathology (and method of discovery) observed amongst trial participants to the research team on a continuous basis during the study, including through each follow-up survey. Adverse event reports will be reviewed by the DMC (detailed below).

### Data monitoring committee

The independent DMC includes an independent statistician, an independent senior researcher and an independent academic GP. The DMC will monitor aspects of the trial related to ethics, safety and data integrity. Because intervention efficacy analysis cannot be conducted until 6 months post-intervention and the total recruitment period is anticipated to be approximately 11 months, there will not be any potential for early termination of the study based on favourable or unfavourable results from interim analyses. Furthermore, cluster randomised trials are not suited to interim analysis because study power is contingent largely on the number of clusters (GPs) recruited at any point in time [38, 39]; given that this study will be recruiting patients from clusters (GPs) in sequence, there will not be sufficient data for interim analysis. Consequently, no interim analyses will be performed.

If serious adverse events are reported to the DMC which are unexpected and potentially related to the intervention, the DMC will be able to break the randomisation code to assess if the participant was part of the intervention or control group. The DMC will be able to provide this information to those providing health care to the participant and will determine whether there are grounds for stopping the trial early.

### Measures

**Fig. 3:**
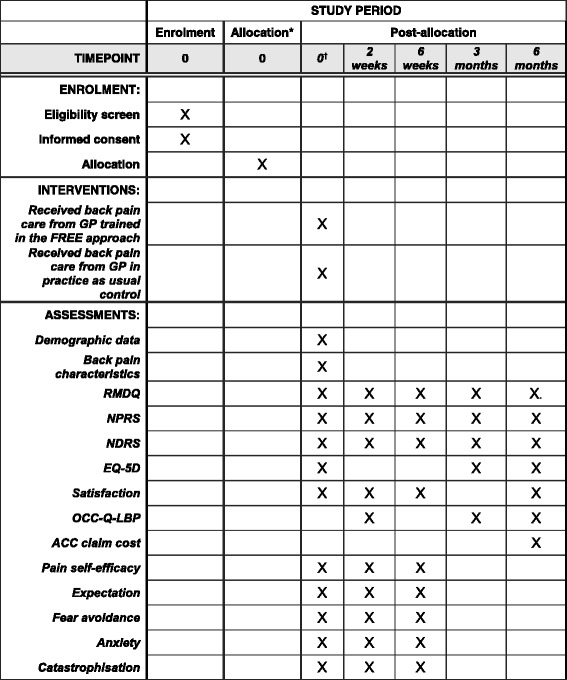
Standard Protocol Items: Recommendations for Interventional Trials (SPIRIT) figure for patient participants. *Patient participants were assigned to intervention/control on the basis of allocation of the practice to which they presented (practices were randomized in this cluster randomized trial). ^†^Assessments completed prior to general practitioner consultation and intervention or control exposure. *RMDQ* Roland-Morris Disability Questionnaire, *NPRS* Numeric Pain Rating Scale, *NDRS* Numeric Disability Rating Scale, *EQ-5D* EuroQol five dimensions, *OCC-Q-LBP* Otago Costs and Consequences Questionnaire, *ACC* Accident Compensation Corporation, *FREE* Fear Reduction Exercised Early, *GP* General practitioner

**Fig. 4:**
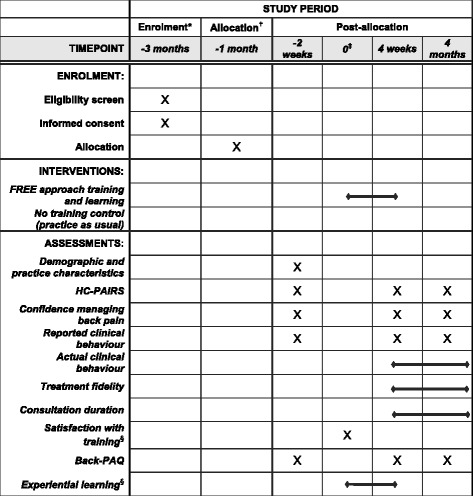
Standard Protocol Items: Recommendations for Interventional Trials (SPIRIT) figure for general practitioner participants. *General practitioners were enrolled between 4 weeks and 1 day pre-randomisation. ^†^Allocation occurred between 5 weeks and 33 weeks before training was received. ^‡^Time point when intervention group practices attended initial training workshops. ^§^Assessment completed by intervention group participants only. *FREE* Fear Reduction Exercised Early, *HC-PAIRS* Health Care Providers’ Pain and Impairment Relationship Scale, *Back-PAQ* Back Pain Attitudes Questionnaire

Patient-related measures are presented in Fig. 3 (SPIRIT figure patient participants; further details in Additional file 4). GP-related measures are presented in Fig. 4 (SPIRIT figure GP participants; further details in Additional file 5). Patient data will be collected through surveys (electronic or postal); GP data will be collected through surveys (electronic or postal), research nurse audio recording of consultations and audit of clinical notes.

#### Patient self-reported outcomes

The primary patient-level outcome measure will be back-related functional impairment 6 months post-initial LBP consultation (interim data at 2 weeks, 6 weeks and 3 months), measured with the Roland-Morris Disability Questionnaire (RMDQ) [40]. Secondary patient outcomes are listed in Additional file 4. The RMDQ, Numeric Pain Rating Scale (NPRS), satisfaction questions [41, 42] and EuroQol five dimensions (EQ-5D) [43] are validated tools recommended as standardised outcome measures for LBP research [42, 44] which gather data for the three core outcome domains recommended for clinical trials of non-specific LBP: physical functioning (RMDQ), pain intensity (NPRS) and health-related quality of life (EQ-5D) [44].

#### Patient objective outcomes

Secondary objective outcomes are the number of days off from work, the number of days on restricted duties and the quantity of medication taken. These will be self-reported by the patient and collected with the OCC-Q-LBP [45]. Cost-of-care data independently collected by ACC as part of standard back pain claim processing will be used to conduct a sensitivity analysis of OCC-Q-LBP data.

#### Patient-reported process measures

To examine the mechanisms by which FREE might impact patient outcomes, we will collect several patient-reported measures of pain and psychosocial function [46–51] (Additional file 4). These are modifiable factors known to be predictive of disability outcomes for LBP, and they form the targets of the FREE approach (*see* Additional file 3). These measures are collected for use as covariates for adjusting main outcome analysis and to inform mediation analysis [52]. Some questions have been modified for consistency (use of first person in questions for self-report, and Likert scales all set to a 7-point range).

#### GP self-reported outcomes

GP-level self-reported outcomes include beliefs about pain and impairment [53, 54], GP confidence [55] and reported clinical behaviour [56] (Additional file 5).

#### GP objective outcomes

GP-level objective outcomes will be GP consultation content recorded from EMR consultation notes by the research nurse immediately after consults and patient report of GP behaviour.

#### GP process measures

To examine the mechanisms by which FREE might impact GP behaviour, we will collect measures related to GP learning [57], satisfaction with training, treatment fidelity and proposed mechanisms of impact (Additional file 5) [58]. Intervention group GPs will record the number of FREE consultations they conduct during the experiential learning period. Associations between consultation count and GP knowledge, beliefs, behaviour, confidence and patient outcome will be analysed to explore whether a minimum number of training consultations is required to become competent in the approach.

Consultation recordings will be analysed to explore whether FREE consultations are longer than usual care LBP consultations. One consultation recording will be randomly selected from each GP with audio recordings available for content and thematic analysis. All consultations selected will be analysed using the Consult Audio Recording Checklist. The researcher conducting this analysis will be blind to group allocation.

### Data management

Study data will be collected and managed using REDCap (Research Electronic Data Capture) tools hosted at the University of Otago [59]. REDCap is a secure, web-based application designed to support data capture for research studies.

### Statistical analysis

Statistical significance will be judged with an α of 0.05. All results will be reported as estimates of effect size (e.g., mean difference, risk ratio, number needed to treat [NNT]) with 95% CI. All analyses will be conducted on an intention-to-treat basis.

Analyses will also account for clustering of data as appropriate. For the patient-level outcomes, this clustering will be specified at the GP level using random effects specifying the GP. Whereas randomisation is at the practice level, clustering effects on outcome values are expected to be driven mostly by inter-practitioner variability. For the GP-level outcomes, clustering will be specified at the practice level. The stratified randomisation will be handled by including the practice size stratum identifier in analytical models (less than or equal to eight FTE GPs, more than eight FTE GPs).

In most instances, analysis will be conducted using linear mixed models (for continuous numerical outcomes such as the RMDQ) or generalised linear mixed models (for categorical outcomes such as satisfaction levels). These models allow for inclusion of data from between baseline and the final endpoint to better estimate the differences at the main endpoint in the presence of missing follow-up data [60]. These models will be adjusted for important baseline covariates (age, sex, socioeconomic status, current back pain duration and nature [constant or episodic], receipt of recent or ongoing non-GP health care for back pain, previous history of back pain, baseline disability and baseline psychological factors [pain self-efficacy and recovery expectations]) [61]. The analysis will also be adjusted for baseline Health Care Providers’ Pain and Impairment Relationship Scale (HC-PAIRS) scores as measured at the level of the GP (as a measure of baseline treatment competence). We will analyse categorical outcomes using generalised linear mixed models (conditional treatment estimates) and generalised estimating equations (marginal treatment estimates) for each outcome: The former approach is to be considered the primary analysis for that output because the conditional treatment effect estimates relate to expected gains for each patient [60, 62].

For the primary outcome, we will report the relative risk (RR) of a 30% improvement in RMDQ score along with the NNT to achieve this target [63]. As a sensitivity analysis, the NNT and RR of a 2.5-point absolute reduction in RMDQ score will also be reported (in line with the absolute effect size stipulated for the primary outcome).

#### Non-inferiority criterion

If the lower bound of the 95% CI for the RMDQ mean difference sits above −2.5 points (the minimal clinically important difference), then we will take this as evidence that the intervention is no worse than current management.

### Patient cost-utility analysis

By performing a cost-utility analysis, we will estimate the mean incremental cost per quality-adjusted life-year (QALY) gained (with QALYs to be calculated with the NZ EQ-5D Tariff 2, as recommended by the New Zealand Pharmaceutical Management Agency [64, 65]) from health care system, ACC and societal perspectives. Reference costs will be assigned for (1) all health care items to allow direct comparison and decrease patient recall requirements, (2) paid work (based on the mean income for someone of the participant's age and gender) to avoid data being skewed by participants with high income and to make results more generalisable to the general population, and (3) unpaid/voluntary work (based upon the minimum wage). The human capital approach will be used for work loss. Because each patient will be followed for 6 months, discounting will not be necessary.

We will report the incremental cost-effectiveness ratio and the monetary incremental net benefit with 95% CI around the estimates. Bootstrapped data will be displayed on a cost-effectiveness plane, and cost-effectiveness acceptability curves will be calculated to determine the likelihood that FREE will be considered cost-effective using one, two and three times gross domestic product per capita as policy-relevant willingness-to-pay thresholds. We will report results from sensitivity analyses by varying inputs over a range of feasible estimates for costs or effects of important variables that are likely to have wide variability (e.g., between small towns and large cities, or chance variability introduced by large costs in a single individual in one group).

### Patient process measures

The aim of mediation analysis will be to examine the *pathways* through which the intervention influences outcomes. We will examine how changes in patient beliefs and attitudes (process variables in Additional file 4) from baseline to week 2 mediate the impact of FREE on the primary outcome (RMDQ score). These analyses are being planned using structural equation modelling or structural mean models approaches [66].

### GP outcomes

As per the patient outcomes, all analyses for GP outcomes will be conducted on an intention-to-treat basis. Clustering of responses will be handled by including a random effect for GP practice in analytical models*.* Continuous outcomes (e.g., HC-PAIRS, confidence scores) will be analysed using linear mixed models. Reported clinical behaviour relating to the case vignette (categorical outcome from five options) will be compared in two ways [56]:

1. Changes in response proportions across the entire range of responses will be compared between arms using a generalised linear mixed model with a cumulative logit link function (to allow for ordinal nature of outcome), adjusted for baseline values and including a random effect for GP practice (to handle clustering).
2. Changes in the proportions of consultations following ‘guideline-consistent’ reported behaviour (in contrast to ‘guideline-inconsistent’ behaviour) will be compared between arms using a generalised linear mixed model with a logit link (i.e., akin to a logistic regression specification), with responses categorised into two levels (‘guideline-consistent’ versus ‘guideline-inconsistent’ [56]). This analysis will be adjusted for baseline values and include a random effect for GP practice to handle clustering.

GP behaviour in the initial LBP patient consultation will be compared between intervention and control group GPs using data from patient management systems and patient report (separate analyses for recommendations regarding work, activity and medication, as well as for referrals to physiotherapy/osteopathy/chiropractic, specialist, x-rays or other scans). Summary statistics will be calculated for each GP (e.g., proportion of patients seen by that GP who reported being advised to take time off from work) and compared between study arms using a linear mixed model for the mean percentage of patients meeting each indicator.

### GP process measures

Process evaluation will be undertaken using mixed methods. Both quantitative and qualitative data from a range of participants and from various sources will be analysed. These include the sample of audio-recorded consultations which will be assessed for fidelity to the FREE approach using a checklist based on FREE, EMR data and the patient-reported consultation content, and GP questionnaires. In order for an audio-recorded consultation to be judged as being faithful to the FREE approach, it must include (1) exploration of patient concerns (item 1 from the Audio Recording Checklist), (2) explanation and reassurance (items 4c–4f or item 7) and (3) work and/or activity advice (item 5). The mean duration of FREE approach consultations will be compared with control consultations using linear mixed models (allowing for clustering by GP; no adjustment for any baseline values).

### Adverse events

The number of minor side effects, cases of serious pathology, and serious adverse events will be compared between groups at the end of the study.

### Sample size

For 80% power to detect a between-group difference of 2.5 RMDQ points (the minimal clinically important difference) at 6 months, assuming SD of 6.0 [67] at a *p* value < 0.05 significance level, an individually randomised trial would require 91 patients per group. This has been inflated to 110 patients per group after adjustment for GP cluster effects (assuming a GP intra-cluster correlation coefficient at a conservative 0.05 and, on average, five patients per GP completing the trial). We aim to recruit 275 patient participants to allow for 20% loss to follow-up.

This design requires 22 GPs per study arm. To account for potential zero recruitment by some GPs, we will recruit 30 GPs per study arm. To further account for patient loss to follow-up (assumed ~ 20% of patients per GP) we will ask GPs to recruit seven patients. This gives a maximal potential sample size of 210 patients per study arm if all GPs recruit an average of 7 patients (total sample size = 60 GPs, 420 patients). Sample size calculations were performed using Stata 12 software (StataCorp, College Station, TX, USA).

### Trial governance

This trial has a trial management committee, a broader trial steering committee and an independent DMC. Roles and responsibilities are described in Additional file 6.

### Publication and dissemination

The results of this study will be disseminated regardless of the magnitude or direction of the effect. Results will be presented in four publications:

1. Main outcome report including the primary outcome together with main secondary and economic outcomes
2. Detailed health economic evaluation
3. Detailed process evaluation
4. Quantitative mediation analysis exploring the mechanisms by which FREE might improve patients health

The study results will be provided to the general public and the general medical community through publication in peer-reviewed journals and presentations at conferences. The study results will be provided directly to study participants through email and through the www.lowbackpain.co.nz website. During the trial, this web address will be directed to a site providing study information appropriate for both control and intervention group participants; results will not be published on this site until after the trial has been completed.

## Discussion

A number of tools exist to classify patients presenting with LBP and allocate treatment on the basis of risk of poor outcome [68, 69]. These have been found to have acceptable predictive ability for disability outcomes but poor predictive ability for persistent pain [70]. Encouraging GPs to simply apply these screening tools and plan subsequent referred care on the basis of risk profiles misses the opportunity for a positive therapeutic encounter delivered by the GP at the point on entry into the health system. Even if a GP is to base treatment decisions on a classification process, there is still a need to ensure this first interaction with a health practitioner is delivered in an optimal manner and aids rather than impedes future care. Because FREE is delivered by a GP within normal consultation time, it does not add cost to an episode of care. Therefore, if FREE is found to be effective and does not increase other costs, it may be a cost-effective method of improving LBP care.

In a previous study in which Dutch GPs were trained to explore and address psychosocial factors in patients who had subacute LBP, researchers did not find any clinically relevant benefits [71]. Jellema et al. [72] concluded that this was because GPs were only moderately successful at identifying psychosocial factors and postulated that 20-minute consultations were insufficient for restructuring patients’ dysfunctional beliefs. The FREE approach differs from the intervention of Jellema et al. in three key ways. Firstly, it aims to restructure the basic GP consultation rather than providing it by way of an additional appointment. This makes the approach appear to be standard practice rather than an add-on, allows the earliest possible intervention, minimises the risk of the GP unwittingly increasing patient threat or fear through their usual practice [73], and ensures that a consistent approach can be used in any subsequent encounters. Secondly, FREE bases the exploration of psychosocial factors around the identification and minimisation of factors which increase patient threat in relation to their back pain rather than including all potential psychosocial influences which may overwhelm GPs [33]. Thirdly, it also provides the patient with a booklet and website which support and reinforce the information provided by the GP.

### Limitations

FREE was initially planned to be delivered during the first consultation which someone receives for an episode of acute LBP, and this study was designed to explore the effect of FREE in this population. The study population was expanded to include people with LBP of any duration for two reasons. Firstly, presentation rates of eligible patients to the first recruitment sites was unacceptably low and indicated that recruitment targets would not be met within time frames that were acceptable to either participating practices or the project budget. Secondly, feedback from participating GPs indicated that these restrictive recruitment criteria excluded most of the patients with LBP they commonly saw. Consequently, the generalisability of study findings was likely to be limited. The revised recruitment criteria are similar to those used in previous LBP primary care research [67]. A sensitivity analysis of the main outcomes will be performed restricted to the original study population of participants with acute LBP.

GPs will complete baseline measures post-randomisation (but prior to receiving training). Training requires that all participating GPs in a practice attend a 4-h training session together. Although it would be preferable to have all GPs complete baseline measures prior to randomisation, this is not possible, given the lead-in time required to schedule and co-ordinate training sessions with the practices. GPs will be aware of whether they will receive training, but they will not be aware of the content of the intervention when they complete baseline measures. It is possible that knowledge of group allocation could affect GPs’ responses when completing baseline measures; however, it is considered more important that these be completed close to when training is received and patients are recruited. GP measures are not the trial’s main outcome, and patients are recruited while blind to group allocation.

## Trial status

This is protocol version 2 (amended 8 December 2016). GP participants were recruited between 6 July and 8 August 2016. Patient participant recruitment began on 23 September 2016 and concluded on 31 July 2017.

## Additional files

Additional file 1: SPIRIT checklist. (PDF 277 kb)

Additional file 2: Behaviour change techniques employed in FREE in relation to general practitioners. (PDF 413 kb)

Additional file 3: Behaviour change techniques employed in FREE in relation to patients. (PDF 409 kb)

Additional file 4: Patient-related measures. (PDF 446 kb)

Additional file 5: General practitioner-related measures. (PDF 418 kb)

Additional file 6: Additional trial information (consistent with SPIRIT 2013 checklist) not included in the main protocol document. (PDF 344 kb)

Additional file 7: Exemplar of GP participant information sheet and consent form. (PDF 127 kb)

Additional file 8: Exemplar of patient participant information sheets and consent form. (PDF 173 kb)

## Abbreviations

ACC: Accident Compensation Corporation
Back-PAQ: Back Pain Attitudes Questionnaire
BCT: Behaviour change technique
DMC: Data monitoring committee
EMR: Electronic medical record
EQ-5D: EuroQol five dimensions
FREE: Fear Reduction Exercised Early
FTE: Full-time equivalent
GP: General practitioner
HC-PAIRS: Health Care Providers’ Pain and Impairment Relationship Scale
LBP: Low back pain
LBPinGP: Low Back Pain in General Practice
NNT: Number needed to treat
NDRS: Numeric Disability Rating Scale
NPRS: Numeric Pain Rating Scale
OCC-Q-LBP: Otago Costs and Consequences Questionnaire for Low Back Pain
PHO: Primary healthcare organisation
PI: Principal investigator
QALY: Quality-adjusted life-year
REDCap: Research Electronic Data Capture
RMDQ: Roland-Morris Disability Questionnaire
RR: Relative risk
SPIRIT: Standard Protocol Items: Recommendations for Interventional Trials
WTP: Willingness to pay
