# Supplementary material for: The Fear Reduction Exercised Early (FREE) approach to low back pain: study protocol for a randomised controlled trial
Source: Trials. 2017 Oct 17;18:484. doi: 10.1186/s13063-017-2225-8 (PMC5646107; doi:10.1186/s13063-017-2225-8)
Supplement: Supplementary file 2 — Behaviour change techniques employed in FREE in relation to general practitioners. (PDF 413 kb) [file 13063_2017_2225_MOESM2_ESM.pdf]

Additional file 2. GP behaviour change goals, techniques and assessment These techniques are described using the behaviour change technique taxonomy developed by Michie et al. [1].

GOAL	BEHAVIOUR CHANGE TECHNIQUE*[1]	MODE OF DELIVERY	MEASURE	MEASUREMENT TOOL
Practice the approach enough to become confident	2) Reward and threat<br>• Incentive	GP receives a $20 voucher for each of the first three times they use the approach	• GP claims of vouchers<br>• Confidence in ability to use the approach<br>• Confidence in ability to manage LBP	• Count<br>• (GP interviews implementation study)<br>• Confidence to manage LBP
Use of FREE becomes normal	3) Repetition and substitution<br>• Habit formation	Encouraged to use with all LBP patients	• Proportion LBP patients with whom the approach is used	• Count
FREE is easy to use	4) Antecedents<br>• Restructuring the physical environment	• Medtech advanced form with prompts and guidance<br>• Training booklet with diagrams for providing explanations to patients	• Number of times the approach is used<br>• Patient receives explanation	• Count<br>• Patient report<br>• Audio recordings of consult
Decreased anxiety about pathology and decreased screening	4) Antecedents<br>• Restructuring the social environment	Information about the risk of pathology, risks of screening, sensitivity of flags, influence on outcome	• Red flag questions later in consultation<br>• Reduced investigation referral	• Audio recordings of consult<br>• Notes audit<br>• Patient report<br>• Otago Costs and Consequences Questionnaire<br>• ACC data
Increase use of the FREE approach	5) Associations<br>• Prompt	• Booklet stand<br>• Medtech tool bar icon<br>• Medtech advanced form	• Number of times the approach is used	• Count
Increased consideration of psychosocial factors	7) Natural consequences<br>• Health consequences	Education regarding factors which influence outcome	• Psychosocial questioning and integration into explanations	• Audio recordings of consult
Increased activity prescription	7) Natural consequences<br>• Health consequences	Education regarding benefits and safety of exercise	• Decreased concern regarding pain and injury<br>• Increased activity recommendation	• Health Care Providers' Pain And Impairment Relationship Scale<br>• Clinical behaviour - vignettes<br>• Patient report<br>• Audio recordings of consult
Increased confidence in FREE	8) Feedback and monitoring<br>• Self-monitoring of outcome of behaviour	Record observations of patient responses on notes page of booklet	• Confidence in ability to use FREE<br>• Confidence in ability to manage back pain	• (GP interviews implementation study)<br>• Confidence to manage LBP

From: Darlow B, Stanley J, Dean S, Abbott JH, Garrett S, Mathieson F, Dowell A (2017). The Fear Reduction Exercised Early (FREE) approach to low back pain: protocol for a cluster randomised, parallel-group, superiority trial of patient impairment 6-months post general practitioner consultation.

GOAL	BEHAVIOUR CHANGE TECHNIQUE*[1]	MODE OF DELIVERY	MEASURE	MEASUREMENT TOOL
	8) Feedback and monitoring<br>• Self-monitoring of behaviour	Tick sheet in booklet for each time uses FREE	• Increased awareness of utility	• Count
Increased understanding of differences between current practice and new approach	11) Comparison of behaviour<br>• Modelling of behaviour	Videos of patient consults integrating FREE in workshop and on website	• Confidence in ability to use FREE<br>• Confidence to manage LBP	• (GP interviews implementation study)<br>• Confidence to manage LBP
Increased confidence in FREE	13) Comparison of outcomes<br>• Persuasive argument	Information from credible source (University of Otago)	• Confidence in ability to use FREE<br>• Confidence to manage LBP	• (GP interviews implementation study)<br>• Confidence to manage LBP
Increased understanding of the back and back pain	15) Shaping knowledge<br>• Antecedents	Information about influences (or lack thereof) on back pain and behaviours/ factors which influence outcome	• Knowledge<br>• Clinical behaviour	• Back-Pain Attitudes Questionnaire<br>• Clinical behaviour - vignettes
Positive experiences of using the approach	15) Shaping knowledge<br>• Behavioural experiment	• Try these five questions<br>• Try these repeated movements	• Questions asked<br>• Movements repeated	• Audio recordings of consult
Confident to use approach with patients	15) Shaping knowledge<br>• Instruction how to perform	• Videos of patient consults integrating FREE in workshop<br>• Role plays in workshop<br>• Specific instructions	• Confidence in ability to use FREE<br>• Confidence to manage LBP	• (GP interviews implementation study)<br>• Confidence to manage LBP

Note: some behaviour change techniques will be assessed through qualitative interviews performed as part of a parallel implementation study.

* The numbers in this list relate to the behaviour change techniques included (and their reference numbers) from Michie et al.'s Behaviour Change Technique Taxonomy (v1) of 93 Hierarchically Clustered Techniques based on international consensus.[1]

1. Michie S, Richardson M, Johnston M, Abraham C, Francis J, Hardeman W, Eccles MP, Cane J, Wood CE: The behavior change technique taxonomy (v1) of 93 hierarchically clustered techniques: building an international consensus for the reporting of behavior change interventions. *Ann Behav Med* 2013, 46(1):81-95.

From: Darlow B, Stanley J, Dean S, Abbott JH, Garrett S, Mathieson F, Dowell A (2017). The Fear Reduction Exercised Early (FREE) approach to low back pain: protocol for a cluster randomised, parallel-group, superiority trial of patient impairment 6-months post general practitioner consultation.
