# Supplementary material for: The Fear Reduction Exercised Early (FREE) approach to low back pain: study protocol for a randomised controlled trial
Source: Trials. 2017 Oct 17;18:484. doi: 10.1186/s13063-017-2225-8 (PMC5646107; doi:10.1186/s13063-017-2225-8)
Supplement: Supplementary file 3 — Behaviour change techniques employed in FREE in relation to patients. (PDF 409 kb) [file 13063_2017_2225_MOESM3_ESM.pdf]

Additional File 3. Patient behaviour change goals, techniques and assessment

These techniques are described using the behaviour change technique taxonomy developed by Michie et al. [1].

GOAL	BEHAVIOUR CHANGE TECHNIQUE*[1]	MODE OF DELIVERY	MEASURE	MEASUREMENT TOOL
• Increase movement<br>• Decrease protection<br>• Improve outcome expectation	<u>2) Reward and threat</u><br>• Anticipation of future rewards	Information – if you move more and fear less you will be more likely to recover	• Activity levels<br>• Work participation<br>• Fear avoidance beliefs<br>• Outcome expectation	• (patient interviews - implementation study)<br>• Self-reported days off work<br>• Fear avoidance items<br>• Expectation items
• Increase movement<br>• Decrease fear of movement	<u>3) Repetition and substitution</u><br>• Behavioural rehearsal	Repeated movements	• How often movements are performed<br>• Fear avoidance beliefs	• (patient interviews - implementation study)<br>• Fear avoidance items
• Remember to perform repeated movements	<u>5) Associations</u><br>• Prompt	Tear out sheet in patient information booklet	• How often movements are performed	• (patient interviews - implementation study)
• Increase movement<br>• Decrease fear of movement<br>• Reduce threat associated with LBP	<u>7) Natural consequences</u><br>• Health consequences	Information – risks and benefits of activity; pain education; recovery behaviours	• Activity levels<br>•<br>• Fear avoidance beliefs<br>• Reduced catastrophisation	• (patient interviews - implementation study)<br>• Pain self-efficacy items<br>• Fear avoidance items<br>• Catastrophisation item<br>• Anxiety item
• Increased support from family and employer	<u>7) Social support</u><br>• Practical and emotional	Tear out information sheets for families and employers	• Support received	• (patient interviews - implementation study)
• Confidence in the FREE approach<br>• Confidence in the GP	<u>13) Comparison of outcomes</u><br>• Persuasive argument	Information from credible source (University of Otago, GP)<br>Profiling role and ability of GP	• Satisfaction	• (patient interviews - implementation study)<br>• Satisfaction with information received and care received
• Improve understanding of the back and back pain (resulting in decreased threat and fear, and improved expectation)	<u>15) Shaping knowledge</u><br>• Antecedents	Information about influences (or lack thereof) on back pain and behaviours/factors which influence outcome	• Knowledge	• Pain self-efficacy items<br>• Fear avoidance items<br>• Catastrophisation item<br>• Anxiety item<br>• Expectation items
• Decrease fear of movement	<u>15) Shaping knowledge</u><br>• Behavioural experiment	Repeated movements with GP	• Activity levels<br>• Fear avoidance beliefs	• (patient interviews - implementation study)<br>• Pain self-efficacy items<br>• Fear avoidance items
• Increase movement	<u>15) Shaping knowledge</u><br>• Instruction how to	Exercises to get you started	• Engage in exercises	• (patient interviews - implementation study)

From: Darlow B, Stanley J, Dean S, Abbott JH, Garrett S, Mathieson F, Dowell A (2017). The Fear Reduction Exercised Early (FREE) approach to low back pain: protocol for a cluster randomised, parallel-group, superiority trial of patient impairment 6-months post general practitioner consultation.

GOAL	BEHAVIOUR CHANGE TECHNIQUE*[1]	MODE OF DELIVERY	MEASURE	MEASUREMENT TOOL
	perform			

Note: some behaviour change techniques will be assessed through qualitative interviews performed as part of a parallel implementation study.

* The numbers in this list relate to the behaviour change techniques included (and their reference numbers) from Michie et al.'s Behaviour Change Technique Taxonomy (v1) of 93 Hierarchically Clustered Techniques based on international consensus.[1]

1. Michie S, Richardson M, Johnston M, Abraham C, Francis J, Hardeman W, Eccles MP, Cane J, Wood CE: The behavior change technique taxonomy (v1) of 93 hierarchically clustered techniques: building an international consensus for the reporting of behavior change interventions. *Ann Behav Med* 2013, 46(1):81-95.

From: Darlow B, Stanley J, Dean S, Abbott JH, Garrett S, Mathieson F, Dowell A (2017). The Fear Reduction Exercised Early (FREE) approach to low back pain: protocol for a cluster randomised, parallel-group, superiority trial of patient impairment 6-months post general practitioner consultation.
