# Supplementary material for: The Fear Reduction Exercised Early (FREE) approach to low back pain: study protocol for a randomised controlled trial
Source: Trials. 2017 Oct 17;18:484. doi: 10.1186/s13063-017-2225-8 (PMC5646107; doi:10.1186/s13063-017-2225-8)
Supplement: Supplementary file 4 — Patient-related measures. (PDF 446 kb) [file 13063_2017_2225_MOESM4_ESM.pdf]

Additional file 4 . Patient related outcome measures

TYPE	DOMAIN	INSTRUMENT	DATA COLLECTION TIME POINTS
Primary	Back-related functional impairment	Roland Morris Disability Questionnaire [1]<br>24-item scale. Each item score yes or no, high scores indicate severe impairment	0, 2w, 6w, 3m, 6m
Secondary	Pain	Numeric Pain Rating Scales<br>How bad has your back pain been in the last 24 hours?<br>How bad has your leg pain been in the last 24 hours?<br>Eleven-point numeric rating scales where 0 = no pain, 5 = moderate pain, and 10 = worst possible pain	0, 2w, 6w, 3m, 6m
	Activity limitation	Numeric Disability Rating Scale<br>How much has your back pain limited your daily activities in the last 24 hours?<br>Eleven-point numeric rating scales where 0 = no limitation, 5 = moderate limitation, and 10 = unable to carry out any activities at all	0, 2w, 6w, 3m, 6m
	Health related quality of life	EuroQol-5D [2]<br>Five-item scale with three response options: no problem, moderate problems, severe problems	0, 3m, 6m
	Satisfaction	With information received<br>How satisfied are you with the information you received about your back pain?<br>Where 0 = very dissatisfied and 6 = very satisfied	0, 2w,
		With care received [3] modified by [4]<br>How satisfied are you with the treatment you received for your back pain?<br>Where 0 = very dissatisfied and 6 = very satisfied	0, 2w, 6w, 6m
		Rating of the overall results of care [3]<br>If you had to spend the rest of your life with the symptoms you have right now, how would you feel about it?<br>Where 0 = very dissatisfied and 6 = very satisfied	2w, 6w, 6m
	Economic consequences	Modified Otago Costs and Consequences Questionnaire for Low Back Pain [5] includes:<br>• Work loss due back problem<br>• Medications consumption<br>• Healthcare consumption<br>• Carer / support person time<br>• Out-of-pocket expenses	2w, 3m, 6m

From: Darlow B, Stanley J, Dean S, Abbott JH, Garrett S, Mathieson F, Dowell A (2017). The Fear Reduction Exercised Early (FREE) approach to low back pain: protocol for a cluster randomised, parallel-group, superiority trial of patient impairment 6-months post general practitioner consultation.

TYPE	DOMAIN	INSTRUMENT	DATA COLLECTION TIME POINTS
		ACC claim cost	6m
Process	Pain self-efficacy	PSEQ-2 [6]<br>Please rate how **confident** you are that you can do the following things at present, **despite the pain**<br>I can do some form of work, despite the pain (''work'' includes housework and paid and unpaid work).<br>I can live a normal lifestyle, despite the pain<br>where 0 = not at all confident and 6 = completely confident	0, 2w, 6w,
	Expectation	I think I will be able to do my regular job, without any restrictions, 4 weeks from now [7]<br>I think I will be able to do my regular job, without any restrictions, 3 months from now [8]<br>Where 0 = Unlikely and 6 = Definitely	0, 2w, 6w
	Fear avoidance	Physical activity might harm my back<br>I should not do physical activities which (might) make my pain worse [9, 10]<br>0 = Completely disagree and 6 = Completely agree	0, 2w, 6w
	Anxiety	I feel anxious or worried about my back[10]<br>0 = Not at all and 6 = Quite anxious	0, 2w, 6w
	Catastrophisation	I feel that my back pain is terrible and it's never going to get any better [11]<br>Where 0 = Not at all and 6 = All the time	0, 2w, 6w

From: Darlow B, Stanley J, Dean S, Abbott JH, Garrett S, Mathieson F, Dowell A (2017). The Fear Reduction Exercised Early (FREE) approach to low back pain: protocol for a cluster randomised, parallel-group, superiority trial of patient impairment 6-months post general practitioner consultation.

References

1. Roland M, Morris R: A study of the natural history of back pain. Part I: development of a reliable and sensitive measure of disability in low-back pain. *Spine* 1983, 8(2):141-144.
2. The EuroQol Group: EuroQol - a new facility for the measurement of health-related quality of life. *Health policy (Amsterdam, Netherlands)* 1990, 16(3):199-208.
3. Deyo RA, Battie M, Beurskens AJ, Bombardier C, Croft P, Koes B, Malmivaara A, Roland M, Von Korff M, Waddell G: Outcome measures for low back pain research. A proposal for standardized use. *Spine* 1998, 23(18):2003-2013.
4. Lamb SE, Lall R, Hansen Z, Castelnuovo E, Withers EJ, Nichols V, Griffiths F, Potter R, Szczepura A, Underwood M: A multicentred randomised controlled trial of a primary care-based cognitive behavioural programme for low back pain. The Back Skills Training (BeST) trial. *Health Technol Assess* 2010, 14(41):1-253, iii-iv.
5. Pinto D, Robertson MC, Hansen P, Abbott JH: Good agreement between questionnaire and administrative databases for health care use and costs in patients with osteoarthritis. *BMC Med Res Methodol* 2011, 11:45.
6. Nicholas MK, McGuire BE, Asghari A: A 2-item short form of the Pain Self-efficacy Questionnaire: development and psychometric evaluation of PSEQ-2. *J Pain* 2015, 16(2):153-163.
7. Shaw WS, Pransky G, Patterson W, Winters T: Early disability risk factors for low back pain assessed at outpatient occupational health clinics. *Spine* 2005, 30(5):572-580.
8. Dionne CE, Bourbonnais R, Frémont P, Rossignol M, Stock SR, Larocque I: A clinical return-to-work rule for patients with back pain. *CMAJ* 2005, 172(12):1559-1567.
9. Hart DL, Werneke MW, George SZ, Matheson JW, Wang Y-C, Cook KF, Mioduski JE, Choi SW: Screening for elevated levels of fear-avoidance beliefs regarding work or physical activities in people receiving outpatient therapy. *Phys Ther* 2009, 89(8):770-785.
10. Kent P, Mirkhil S, Keating J, Buchbinder R, Manniche C, Albert HB: The concurrent validity of brief screening questions for anxiety, depression, social isolation, catastrophization, and fear of movement in people with low back pain. *Clin J Pain* 2014, 30(6):479-489.
11. Wideman TH, Hill JC, Main CJ, Lewis M, Sullivan MJ, Hay EM: Comparing the responsiveness of a brief, multidimensional risk screening tool for back pain to its unidimensional reference standards: the whole is greater than the sum of its parts. *Pain* 2012, 153(11):2182-2191.
