# Supplementary material for: The Fear Reduction Exercised Early (FREE) approach to low back pain: study protocol for a randomised controlled trial
Source: Trials. 2017 Oct 17;18:484. doi: 10.1186/s13063-017-2225-8 (PMC5646107; doi:10.1186/s13063-017-2225-8)
Supplement: Supplementary file 5 — General practitioner-related measures. (PDF 418 kb) [file 13063_2017_2225_MOESM5_ESM.pdf]

Appendix 5. GP related measures

TYPE	DOMAIN	INSTRUMENT	DATA COLLECTION TIME POINTS
Outcome	Relationship between pain and impairment	Health Care providers' Pain and Impairment Relationship Scale (HC-PAIRS) [1, 2]<br><br>13 item scale. Each item is scored on a 7-point Likert Scale ranging from 'Completely Disagree' (1 point) to 'Completely Agree' (7 points), resulting in a possible range from 13 to 91 points; higher scores reflect a stronger belief that pain justifies disability and activity limitation.	-2w*, 4w, 4m
	Confidence	Self-confidence in managing back pain patients [3]<br>Four items<br>1. I lack the diagnostic tools or knowledge needed to effectively assess patients with LBP*<br>2. I know exactly what to do to effectively treat patients with LBP<br>3. I am very comfortable treating patients with LBP.<br>4. How well prepared to manage LBP are you now?<br><br>All rated on five point Likert scale where 1 = strongly agree and 5 = strongly disagree, except item 4 where 1 = very well and 5 = very poorly<br>* item 1 is reversed	-2w*, 4w, 4m
	Reported clinical behaviour	Clinical case vignettes of patient with non-specific LBP with three items related to work, physical activity advice [4]<br><br>Categorical outcome with five response options	-2w*, 4w, 4m
	Actual clinical behaviour	Electronic Medical Record notes audit (include investigation referral)<br><br>Consult Record Form	Post each consult with patient participant
		Patient report immediately post appointment<br>Did your doctor recommend or advise any of the following? (yes or no)<br>• Time off work<br>• Reduced hours at work<br>• Medication<br>• Physiotherapy/Osteopathy/Chiropractic<br>• Specialist<br>• X-rays or scans<br>• Blood tests	Post each consult with patient participant
		Items from modified Otago Costs and Consequences Questionnaire (OCC-Q) [5]<br>• Medication consumption<br>• Healthcare consumption	Gathered from patient participants at 2w, 3m, 6m†
Process	Treatment fidelity	Audio recordings – 1 recording randomly selected from each GP with audio available<br><br>Consult Audio Record Checklist	Post each consult with patient participant

From: Darlow B, Stanley J, Dean S, Abbott JH, Garrett S, Mathieson F, Dowell A (2017). The Fear Reduction Exercised Early (FREE) approach to low back pain: protocol for a cluster randomised, parallel-group, superiority trial of patient impairment 6-months post general practitioner consultation.

TYPE	DOMAIN	INSTRUMENT	DATA COLLECTION TIME POINTS
	Consultation duration	Audio recordings of all consults assessed for<br>1. Total consultation length<br>2. Back pain primary complaint addressed during consultation	Post each consult with patient participant
	Satisfaction with training	Workshop evaluation form	0, (immediately after workshop - intervention group only)
	Learning	Back Pain Attitudes Questionnaire (Back-PAQ) [6]<br>34 item scale based upon six themes. Each item is scored on a 5-point Likert scale ranging from 'True' (1 point) to 'False' (5 points), resulting in a possible range from 34 to 170 points; higher scores indicate more unhelpful beliefs, lower scores indicate more helpful beliefs.	-2w, 4w, 4m
	Experiential learning	GP will record<br>1. The number of FREE consultations they conduct during the training period<br>2. The elements of FREE they use in the first three of these consultations<br>3. Their perceptions of the first three of these consultations	0 to 4w (during experiential learning period - intervention group only)
Demographic	Gender, age, ethnicity, history of back pain		-2w*
Practice characteristics	Years' experience, locality of practice		-2w*

* Measure completed before attending initial workshop (for intervention practices) or matched timing for those in paired control group practice.

† Timing relates to patient participant timeline

From: Darlow B, Stanley J, Dean S, Abbott JH, Garrett S, Mathieson F, Dowell A (2017). The Fear Reduction Exercised Early (FREE) approach to low back pain: protocol for a cluster randomised, parallel-group, superiority trial of patient impairment 6-months post general practitioner consultation.

References

1. Rainville J, Carlson N, Polatin P, Gatchel RJ, Indahl A, Volinn E: Exploration of physicians' recommendations for activities in chronic low back pain. *Spine* 2000, 25(17):2210-2220.
2. Sieben JM, Vlaeyen JW, Portegijs PJ, Warmenhoven FC, Sint AG, Dautzenberg N, Romeijnders A, Arntz A, Knottnerus JA: General practitioners' treatment orientations towards low back pain: influence on treatment behaviour and patient outcome. *Eur J Pain* 2009, 13(4):412-418.
3. Smucker DR, Konrad TR, Curtis P, Carey TS: Practitioner self-confidence and patient outcomes in acute low back pain. *Arch Fam Med* 1998, 7(3):223.
4. Evans DW, Foster NE, Underwood M, Vogel S, Breen AC, Pincus T: Testing the effectiveness of an innovative information package on practitioner reported behaviour and beliefs: The UK Chiropractors, Osteopaths and Musculoskeletal Physiotherapists Low back pain ManagemENT (COMPLeMENT) trial [ISRCTN77245761]. *BMC Musculoskelet Disord* 2005, 6(41).
5. Pinto D, Robertson MC, Hansen P, Abbott JH: Good agreement between questionnaire and administrative databases for health care use and costs in patients with osteoarthritis. *BMC Med Res Methodol* 2011, 11:45.
6. Darlow B, Perry M, Mathieson F, Stanley J, Melloh M, Marsh R, Baxter GD, Dowell A: The development and exploratory analysis of the Back Pain Attitudes Questionnaire (Back-PAQ). *BMJ Open* 2014, 4(5).

From: Darlow B, Stanley J, Dean S, Abbott JH, Garrett S, Mathieson F, Dowell A (2017). The Fear Reduction Exercised Early (FREE) approach to low back pain: protocol for a cluster randomised, parallel-group, superiority trial of patient impairment 6-months post general practitioner consultation.
