# Supplementary material for: The Fear Reduction Exercised Early (FREE) approach to low back pain: study protocol for a randomised controlled trial
Source: Trials. 2017 Oct 17;18:484. doi: 10.1186/s13063-017-2225-8 (PMC5646107; doi:10.1186/s13063-017-2225-8)
Supplement: Supplementary file 6 — Additional trial information (consistent with SPIRIT 2013 checklist) not included in the main protocol document. (PDF 344 kb) [file 13063_2017_2225_MOESM6_ESM.pdf]

Additional file 6. Additional trial information

1. World Health Organization Trial Registration Data Set

Data category	Information	Date amended
Primary registry and trial identifying number	anzctr.org.au ANZCTRN: ACTRN12616000888460	
Date of registration in primary registry	6 July 2016	
Secondary identifying numbers	UTN: U1111-1178-7448	
Source(s) of monetary or material support	Accident Compensation Corporation	
Primary sponsor	University of Otago	
Secondary sponsor(s)	NA	
Contact for public queries	BD (ben.darlow@otago.ac.nz), AD (tony.dowell@otago.ac.nz)	
Contact for scientific queries	BD, AD, JS, University of Otago	
Public title	Low Back Pain in General Practice	
Scientific title	The Fear Reduction Exercised Early (FREE) approach to ~~acute~~ low back pain: a cluster randomised, parallel-group, superiority trial of patient impairment 6-months post general practice consultation	Revised 4/01/2017
Countries of recruitment	New Zealand	
Health condition(s) or problem(s) studied	~~Acute~~ low back pain	Revised 4/01/2017
Intervention(s)	Active comparator: the Fear Reduction Exercised Early (FREE) approach to low back pain	
	Control comparator: practice as usual	
Key inclusion and exclusion criteria	• GPs: Ages eligible for study: all; Sexes eligible for study: both; Accepts healthy volunteers: yes<br>• ~~Patients: Ages eligible for study: 18 to 65 years; Sexes eligible for study: both; Accepts healthy volunteers: no~~<br>• Patients: Ages eligible for study: 18 years -and over-years; Sexes eligible for study: both; Accepts healthy volunteers: no	New criteria for patients 4/01/2017
	Inclusion criteria:<br>• GPs: New Zealand registered GP, working within the trial region, working in a consenting practice<br>• ~~Patients: 18 to 65 years, present to general practitioner with new episode of acute low back pain (<6 weeks duration)~~<br>• Patients: 18 years and over, present to general practitioner with low back pain ~~(<6 weeks~~ any duration)	New criteria for patients 4/01/2017
	Exclusion criteria:<br>• GPs: Have participated in pilot testing<br>• ~~Patients: Have received consultation or treatment for this episode or for other LBP in the previous 3 months, unable to read and write in English~~<br>• Patients: Have had back surgery in the last 6 months, have been unable to do their normal work because of back pain for more than three of the last 6 months, unable to read and write in English	New criteria for patients 4/01/2017

From: Darlow B, Stanley J, Dean S, Abbott JH, Garrett S, Mathieson F, Dowell A (2017). The Fear Reduction Exercised Early (FREE) approach to low back pain: protocol for a cluster randomised, parallel-group, superiority trial of patient impairment 6-months post general practitioner consultation.

Data category	Information	Date amended
Study type	Interventional	
	Allocation: cluster randomized; Intervention model: parallel assignment; Masking: double blind (investigator and patient, not GP)	
	Primary purpose: impairment reduction	
	Phase III	
Date of first enrolment	June 2016	
Target sample size	GPs: 60, Patients: 275	
Recruitment status	Completed	Revised 31/07/2017
Primary outcome(s)	Patient back pain related impairment measured with the Roland Morris Disability Questionnaire (time frame: 6 months; not designated as safety issue)	
Key secondary outcomes	Patient pain intensity and quality of life; GP attitudes and confidence	

From: Darlow B, Stanley J, Dean S, Abbott JH, Garrett S, Mathieson F, Dowell A (2017). The Fear Reduction Exercised Early (FREE) approach to low back pain: protocol for a cluster randomised, parallel-group, superiority trial of patient impairment 6-months post general practitioner consultation.

2. Trial roles and responsibilities

Principal investigator

Dr Ben Darlow

- Design and conduct of the trial
- Preparation of protocol and revisions
- Preparation of trial documentation
- Organising steering committee meetings
- Publication of study reports

Trial Steering Committee

Dr Ben Darlow, Prof Antony Dowell, Dr James Stanley, Assoc Prof. Sarah Dean, Assoc Prof Haxby Abbott, Fiona Mathieson, Sue Garrett

- Agreement of final protocol
- Assistance with independent ethics committee applications (HDEC)
- Reviewing progress of study and if necessary agreeing changes to the protocol and/or trial documentation to facilitate the smooth running of the study.

Trial Management Committee

Dr Ben Darlow, Prof Antony Dowell, Dr James Stanley, Sue Garrett, Freya Morris-Cole

- Study planning
- Organisation of Trial Steering Committee meetings
- Recruitment
- SUSAR [Serious unexpected suspected adverse events] reporting to DMC and HDEC
- Responsible for trial master file
- Budget administration and contractual issues

Trial statistician

Dr James Stanley

- Data management oversight
- Statistical analysis
- Production of outputs of results for dissemination

Independent statistician

Dr Dalice Sim

- Randomisation and storage of key to the randomisation code
- Take the place of the Trial Statistician at Trial Steering Committee or Trial Management Committee meetings if there is a risk that discussions could unblind the Trial Statistician
- Member of the Data Monitoring Committee

Data manager

Sue Garrett

- Maintenance of trial IT system and data entry processes
- Data verification

From: Darlow B, Stanley J, Dean S, Abbott JH, Garrett S, Mathieson F, Dowell A (2017). The Fear Reduction Exercised Early (FREE) approach to low back pain: protocol for a cluster randomised, parallel-group, superiority trial of patient impairment 6-months post general practitioner consultation.

Data Monitoring Committee

Professor Diana Sarfati (independent senior researcher and DMC Chair), Dr Lynn McBain (independent academic general practitioner), Dr Dalice Sim (independent statistician)

- Monitor aspects of the trial related to ethics, safety and data integrity
- Consider SUSAR reports and the need to break the randomisation code

3. Protocol amendments

Any modifications to the protocol which may impact on the conduct of the study, potential benefits to GPs or patients, or patient safety including changes in study objectives, study design, patient population, sample sizes, or study procedures will require a formal protocol amendment. This amendment will be agreed upon by the Trial Management Committee and Trial Steering Committee and approved by the HDEC prior to implementation.

Administrative changes to the protocol are defined as minor corrections and/or clarifications which do not affect study conduct. These will be agreed upon by the Trial Management Committee. The HDEC will be notified at the Trial Management Committee's discretion.

4. Access to data

All member of the Trial Steering Committee will have full access to the final trial dataset.

5. Authorship

Authorship eligibility will be based on fulfilling International Committee of Medical Journal Editors' Criteria (http://www.icmje.org/recommendations/browse/roles-and-responsibilities/defining-the-role-of-authors-and-contributors.html). All Trial Steering Committee members will have the opportunity to contribute to all publications and presentations in a manner which meets these criteria. Additional authors may be included in publications with the approval of the PI or co-PI.

The lead author for the protocol and the primary outcome paper will be the PI. The lead author for other publications and presentations will be determined based on contribution. A proposed author order will be provided when topics are suggested for publication or presentation and this will be reviewed and approved by the PI and co-PI following production of the draft or abstract.

6. Protocol

No later than 1 year after the collection of 6-month follow-up data from all participants the full protocol will be posted on the lowbackpain.co.nz website.

No later than 3 years after the collection of 6-month follow-up data from all participants the completely de-identified data set will be delivered to an appropriate data archive for sharing purposes.

From: Darlow B, Stanley J, Dean S, Abbott JH, Garrett S, Mathieson F, Dowell A (2017). The Fear Reduction Exercised Early (FREE) approach to low back pain: protocol for a cluster randomised, parallel-group, superiority trial of patient impairment 6-months post general practitioner consultation.
