# Supplementary material for: The Fear Reduction Exercised Early (FREE) approach to low back pain: study protocol for a randomised controlled trial
Source: Trials. 2017 Oct 17;18:484. doi: 10.1186/s13063-017-2225-8 (PMC5646107; doi:10.1186/s13063-017-2225-8)
Supplement: Supplementary file 7 — Exemplar of GP participant information sheet and consent form. (PDF 127 kb) [file 13063_2017_2225_MOESM7_ESM.pdf]

LBP IN GP STUDY

UNIVERSITY of OTAGO Te Whare Wānanga o Otāgo NEW ZEALAND

Low Back Pain in General Practice Study

GP Information Sheet

A new approach to managing acute low back pain (LBP) is being implemented in your PHO. Not all practices can be trained at once. To assess if there are any benefits from using the new approach data will be gathered from some practices using the new approach and some practices still practising as usual. Practices taking part in this study will be randomly allocated to receive the new approach in either the first or second wave of training.

Taking part

Taking part is your choice and you can ask for more time to decide. If you don't want to take part, you don't have to give a reason, and it won't adversely affect you. If you do want to take part now, but change your mind later, you can pull out of the study at any time. Participating in the study does not mean that you commit to using the new approach.

What is this study about?

LBP is a significant and costly health condition This study aims to find out if receiving training in the new approach alters GP views and management of acute LBP, as well as whether it alters patient impairment, satisfaction, and costs.

What will I have to do?

You will be asked to complete a survey when you enter the study. You will then be asked to complete surveys again four weeks and four months later. You will also be asked if your appointments with patients participating in the trial can be audio-recorded (with their consent).

Principal investigator:	Co-investigator:
**Dr Ben Darlow**	**Prof Tony Dowell**
Senior Lecturer	Professor of Primary Health Care
Ph. 04 918 6051	Ph. 04 385 5536
Email: ben.darlow@otago.ac.nz	Email: tony.dowell@otago.ac.nz

Department of Primary Health Care and General Practice
University of Otago, Wellington

What are the benefits and risks of this study?

Back pain is a very common problem in general practice and many patients have a slow recovery or develop persistent problems. In addition, previous research has demonstrated that many GPs do not feel that current treatment guidelines are easily integrated into practice. This study will test a new approach which has addressed many concerns raised by patients and GPs, as well as the latest research evidence. You will not receive details of the approach until your training date is booked (so that usual practice is not affected in second wave practices). The approach was very positively received by GPs during pilot testing.

There are no known risks to taking part. You will be offered a $20 voucher after completing each of the three surveys ($60 total). You will also be able to claim CME credits for time spent participating in this research as Additional Professional Development Activities.

Practices allocated to the first wave will receive training in the new approach before all other practices in the PHO. Practices allocated to the second wave will receive training as soon as possible without jeopardising the results of the study; this is likely to be before many other PHO practices. When you receive training you will be able to claim four CME credits and invoice the University for $800 to compensate for your time.

Who can take part?

GPs working in general practices in the Hutt Valley which have more than three full time equivalent GPs are eligible to take part unless they have already been trained in the new approach during its development.

General information

This study is based in the Department of Primary Health Care and General Practice at the University of Otago, Wellington. It will run over two years.

If you require more information about the study please feel free to contact Ben Darlow or Tony Dowell (details on Page 1). If you have any queries or concerns regarding your rights as a participant in this study, you may wish to contact your professional organisation or an independent health and disability advocate:

Free phone: 0800 555 050, Free fax: 0800 2 SUPPORT (0800 2787 7678)

Email: advocacy@hdc.org.nz

You can also contact the health and disability ethics committee (HDEC) that approved this study on: 0800 4 38442 (0800 4 Ethic) or email: hdecs@moh.govt.nz

Confidentiality

Findings from this study will be published in academic journals and presented at conferences. Nothing that could personally identify you will be used in any reports on this study. You will be allocated a reference code when you enrol in this study, and this code (not your name) will be recorded on all of your documents and consult recordings. The only form that will have both your name and your code will be the Consent Form.

During the study the Consent Form will be securely stored in the Department of General Practice at the University of Otago, Wellington. The remainder of the information and consult recordings, referred to only by your code, will be securely stored by the researchers. You have the right to access information about you collected as part of this study The information collected as part of this study will be stored by the University for ten years. Study information may also be used for future research but you will not be identified in any of this information.

Risks to patients

There are no known risks to patients receiving standard LBP care or the new approach. If you think that your patient has experienced an adverse event from LBP care, please contact the research team. Please also contact the research team if the back pain of any patients taking part in the trial is found to be due to pathology (e.g. infection, tumour, cauda equine, abdominal aortic aneurysm).

How can you find out about the results?

Results of this study will be posted on www.lowbackpain.co.nz. Please note that there may be a significant delay between receiving training and the publication of the results. It is expected results will be available in 2018.

Funding

The development of the new approach was funded by Lotteries Health. The implementation of the new approach and the evaluation study is being funded by the Accident Compensation Corporation (ACC). Study analysis will be conducted independent of ACC.

Ethical approval

This study has received ethical approval from the National Health and Disability Ethics Committee, ethics reference number 16/CEN/43.

Please feel free to contact the researchers if you have any questions about this study

Low Back Pain in General Practice

Consent Form for Participation

	YES	NO
1. I have read and understood the information sheet dated 17/06/2016. I have had the opportunity to ask questions and I am happy with the answers I have received. I freely agree to take part in this study.	☐	☐
2. I agree to have acute LBP consultations I conduct during the course of this study audio-recorded (with patient consent).	☐	☐
3. I agree that the researchers can access clinical notes related acute LBP consultations I conduct during the course of this study (with patient consent).	☐	☐

PLEASE PRINT CLEARLY

I ______________________________ agree to take part in this study.
first name *surname*

Signed: ______________________ Date: ______________

Practice Address: ______________________________

____

______________________________ Post code: ____________

Phone: Home ____________ Work ____________ Cell ____________

Email: ______________________________

Alternative email: ______________________________

Preferred way to receive follow-up surveys:

Post ☐ Email web link ☐ Email document ☐

Researcher:	Ben Darlow
Contact phone number for researcher:	04 9186051 021 0794693
Project explained by:	
Project role:	
Signature:	
Date:	
Participant ID number:	
