# Supplementary material for: The Fear Reduction Exercised Early (FREE) approach to low back pain: study protocol for a randomised controlled trial
Source: Trials. 2017 Oct 17;18:484. doi: 10.1186/s13063-017-2225-8 (PMC5646107; doi:10.1186/s13063-017-2225-8)
Supplement: Supplementary file 8 — Exemplar of patient participant information sheets and consent form. (PDF 173 kb) [file 13063_2017_2225_MOESM8_ESM.pdf]

Low Back Pain in General Practice

Low Back Pain in General Practice Study

Patient Information Summary Sheet

- This study is being run by the University of Otago, Wellington
- It aims to understand how doctors manage low back pain in general practice and how people recover from low back pain
- Findings will help show the best way for doctors to treat people who have low back pain
- Taking part is your choice and you can pull out of the study at any time
- If you take part you will be asked to:
    - **Complete a survey about how your back pain is affecting you before your appointment and answer some questions after**
    - **Decide if your appointment can be audio-recorded to look at how your doctor asks questions and explains back pain**
    - **Decide if your clinical notes related to your back pain can be viewed by researchers**
    - **Complete surveys in two weeks', six weeks', three months, and six months' time to record how your back pain affects your life, the costs associated with it, and the help you have to manage it**
- You will be given $20 vouchers after completing the surveys
- There are no known risks from taking part in this study
- This study will not affect the treatment you receive
- Findings from this study will be published but nothing that could identify you will be used in any reports
- This study has been approved by the Central Health and Disability Ethics Committee (16/CEN/43)

Low Back Pain in General Practice Study

Patient Information Sheet

You are invited to take part in a study about low back pain. Low back pain is a very common reason to visit your doctor. It is important to know how doctors manage low back pain in general practice, what people think about the care they receive and how well they recover. This will help to improve the care people receive for back pain and how this care is provided.

Taking part

Taking part is your choice and you can ask for more time to decide. If you don't want to take part, you don't have to give a reason, and it won't affect the care you receive. If you do want to take part now, but change your mind later, you can pull out of the study at any time.

This information sheet will help you to decide if you'd like to take part. If you decide to take part you will be asked to sign a consent form. You may keep this information sheet.

What is this study about?

This study will find out what happens when someone sees a doctor about back pain. Findings will help show the best way for doctors to treat people who have low back pain.

What will I have to do?

You will be asked to provide contact details and fill out some surveys before you see the doctor today, and answer a few questions straight after your appointment. You will also be asked if your appointment can be audio-recorded (to look at how your doctor asks questions and explains back pain) and if researchers can access your clinical notes related to your back pain. You will then be asked to complete surveys in two weeks, six weeks, three months and six months' time. As some of the questions asked in these surveys relate to the cost of your back pain, you will be a given a log book to record these in to aid your memory.

Principal investigator:
Dr Ben Darlow
Senior Lecturer
Ph. 04 918 6051
Email: ben.darlow@otago.ac.nz

Co-investigator:
Prof Tony Dowell
Professor of Primary Health Care
Ph. 04 385 5536
Email: tony.dowell@otago.ac.nz

Department of Primary Health Care and General Practice
University of Otago, Wellington

What are the benefits and risks of this study?

You will be given a $20 voucher after completing each of the first four surveys ($80 total) and entered in a prize draw for either a two night Air New Zealand Mystery Escape for two, or an $850 Prezzy® Card when you return your final survey. You will not receive any extra treatment benefit from taking part in this study.

There are no known risks to taking part. If you think that you have become worse as a result of the treatment you receive, please contact your GP and the researchers (details on Page 1).

Who can take part?

All people aged 18 years and over who have low back pain. Unfortunately you cannot take part if you have had back surgery in the last six months, if you have been unable to do your normal work because of your back pain for more than three of the last six months, or if you cannot read and write in English.

General information

No one will be told you are taking part in this study unless you ask us to. Your normal health care will not be affected by this study.

You may have a friend, family or whānau support to help you understand this study or the risks and benefits of taking part. You do not have to answer all of the questions in the survey and you can stop the survey at any time.

This study is based in the Department of General Practice at the University of Otago, Wellington. It will run over two years.

If you have any questions, concerns or complaints about the study at any stage, you can contact Ben Darlow or Tony Dowell (details on Page 1).

If you want to talk to someone who isn't involved with the study, you can contact an independent health and disability advocate on:

Free phone: 0800 555 050
Free fax: 0800 2 SUPPORT (0800 2787 7678)
Email: advocacy@hdc.org.nz

You can also contact the health and disability ethics committee (HDEC) that approved this study on: 0800 4 38442 (0800 4 Ethic) or email: hdecs@moh.govt.nz

Privacy

Findings from this study will be published in academic journals and presented at conferences. Nothing that could identify you will be used in any reports on this study. You will be given a reference code when you join the study, and this code (not your name) will be recorded on all of your documents and consult recordings. The only form that will have both your name and your code will be the Consent Form.

During the study the Consent Form will be securely stored in the Department of General Practice at the University of Otago, Wellington. The rest of the information and consult recording (referred to only by your code) will be securely stored by the researchers. You have the right to access information about you collected as part of this study The information collected as part of this study will be stored by the University for ten years. Study information may also be used for future research but you will not be identified in any of this information.

How can you find out about the results?

Results of this study will be posted on www.lowbackpain.co.nz. Please note that there may be a long delay between your doctor's appointment and the publication of the results. It is expected results will be available in 2018.

Funding

This study is being funded by the Accident Compensation Corporation (ACC). ACC will not influence your treatment as a result of this study. Study analysis will be conducted independent of ACC.

Ethical approval

This study has received ethical approval from the Central Health and Disability Ethics Committee, ethics reference number 16/CEN/43.

Please feel free to contact the researchers if you have any questions about this study

Low Back Pain in General Practice
Consent Form for Participation

	YES	NO
1. I have read and understood the information sheet dated 07/12/2016. I have had the opportunity to ask questions and I am happy with the answers I have received. I freely agree to take part in this study.	☐	☐
2. I agree that my back pain appointment may be audio-recorded. I understand that I can ask for this recording to be deleted at any time without explaining	☐	☐
3. I agree that the researchers can access information related to my back pain from my doctor's notes.	☐	☐
4. I agree that the researchers can update my contact details using information from my medical practice during the course of the study.	☐	☐
5. I agree that the researchers can try to contact me again in two and five years' time to request I complete additional surveys related to this study.	☐	☐

PLEASE PRINT CLEARLY

I ____ agree to take part in this study.
first name *surname*

Signed:______________________________ Date:____________________

Address:____

____

____________________________________ Post code:_________________

Phone: Home _______________ Work _______________ Cell _________________

Email: ____

Alternative email: ____

Social media names (e.g. Facebook, Twitter): ____________________________________

Alternative contact person (e.g. friend/relative) in case we cannot get hold of you:

Name/Phone: ____

Preferred way to receive follow-up surveys: **Post** ☐ **Web link (sent by email)** ☐

What type of gift vouchers would you like to receive to acknowledge your study participation? (*you are eligible for a $20 gift voucher after completing each of the first four surveys*):

New World voucher ☐ **MTA (petrol) voucher** ☐ **I would not like to have a voucher** ☐

Researcher:	Ben Darlow
Contact phone number for researcher:	04 9186051
Project explained by:	
Project role:	
Signature:	
Date:	
Participant ID number:	____-____ ____-_____
